# The relationship of Asperger’s syndrome to autism: a preliminary EEG coherence study

**DOI:** 10.1186/1741-7015-11-175

**Published:** 2013-07-31

**Authors:** Frank H Duffy, Aditi Shankardass, Gloria B McAnulty, Heidelise Als

**Affiliations:** 1 Department of Neurology, Boston Children’s Hospital and Harvard Medical School, 300 Longwood Avenue, Boston, Massachusetts 02115, USA; 2 Department of Psychiatry (Psychology), Boston Children’s Hospital and Harvard Medical School, 300 Longwood Avenue, Boston, Massachusetts 02115, USA

**Keywords:** Asperger’s syndrome, Autism spectrum disorder, Connectivity, Discriminant function analysis, EEG, GMM, Mixture modeling, Pervasive developmental disorder-not otherwise specified, PDD-nos, PCA, Principal components analysis, Spectral coherence

## Abstract

**Background:**

It has long been debated whether Asperger’s Syndrome (ASP) should be considered part of the Autism Spectrum Disorders (ASD) or whether it constitutes a unique entity. The Diagnostic and Statistical Manual, fourth edition (DSM-IV) differentiated ASP from high functioning autism. However, the new DSM-5 umbrellas ASP within ASD, thus eliminating the ASP diagnosis. To date, no clear biomarkers have reliably distinguished ASP and ASD populations. This study uses EEG coherence, a measure of brain connectivity, to explore possible neurophysiological differences between ASP and ASD.

**Methods:**

Voluminous coherence data derived from all possible electrode pairs and frequencies were previously reduced by principal components analysis (PCA) to produce a smaller number of unbiased, data-driven coherence factors. In a previous study, these factors significantly and reliably differentiated neurotypical controls from ASD subjects by discriminant function analysis (DFA). These previous DFA rules are now applied to an ASP population to determine if ASP subjects classify as control or ASD subjects. Additionally, a new set of coherence based DFA rules are used to determine whether ASP and ASD subjects can be differentiated from each other.

**Results:**

Using prior EEG coherence based DFA rules that successfully classified subjects as either controls or ASD, 96.2% of ASP subjects are classified as ASD. However, when ASP subjects are directly compared to ASD subjects using new DFA rules, 92.3% ASP subjects are identified as separate from the ASD population. By contrast, five randomly selected subsamples of ASD subjects fail to reach significance when compared to the remaining ASD populations. When represented by the discriminant variable, both the ASD and ASD populations are normally distributed.

**Conclusions:**

Within a control-ASD dichotomy, an ASP population falls closer to ASD than controls. However, when compared directly with ASD, an ASP population is distinctly separate. The ASP population appears to constitute a neurophysiologically identifiable, normally distributed entity within the higher functioning tail of the ASD population distribution. These results must be replicated with a larger sample given their potentially immense clinical, emotional and financial implications for affected individuals, their families and their caregivers.

## Background

Autism or Autism Spectrum Disorder (ASD) is one of the most common neurodevelopmental disorders, with an estimated incidence of 1 in 88 children [1]. According to the Diagnostic and Statistical Manual of Mental Disorders, fourth edition (DSM-IV), a diagnosis of ASD requires the fulfillment of a minimum of six behavioral diagnostic criteria from the following three domains: at least two symptoms of impairment of social interaction, at least one symptom of impairment in communication, and at least one symptom of restricted repetitive and stereotyped patterns of behavior [2]. Moreover, ASD requires symptoms of delay or abnormal functioning with onset prior to age 3 years in at least one of the following three domains: social interaction, language as used in social communication, and symbolic or imaginative play.

In order to establish a diagnosis of Asperger’s syndrome (ASP) [3-6], the DSM-IV requires, as for ASD, the fulfillment of at least two symptoms of impaired social interaction and at least one symptom of restricted, repetitive behavior. However, the ASP diagnosis, in contrast to the ASD diagnosis, does not require a symptom of impairment in communication, nor must any of the symptoms show an onset before age 3 years. According to the DSM-IV, ‘Asperger’s Disorder can be distinguished from Autistic Disorder by the lack of delay in language development. Asperger’s Disorder is not diagnosed if criteria are met for Autistic Disorder’ [2]. Data for the prevalence of ASP are not reliably available, owing to the use of slightly differing diagnostic criteria in the literature. For example, Mattila *et al*. [7] applied four different criteria on the same group of 5,484, eight-year-old children and found prevalence rates varying from 1.6 to 2.9 per 1,000. Kopra *et al*. [8] similarly compared various diagnostic criteria and concluded that ‘the poor agreement between these sets of diagnostic criteria compromises comparability of studies (of Asperger’s syndrome)’.

The specificity of the DSM-IV diagnostic criteria and the classification of ASP as a separate entity have been reconsidered by the Neurodevelopmental Disorders Work Group, resulting in a redefinition of diagnostic boundaries. In the new DSM-5, ASP falls into ASD with essential equivalence to high functioning autism (HFA) and the ‘Asperger’s Syndrome’ name has been dropped [9]. Although clearly intended as a reasonable nosological correction, it places children with severe autism, who have significantly impaired language and/or interaction capacities, under the same ASD umbrella as those who have milder forms, such as HFA and ASP, who lack social skills yet possess normal to high intelligence and typically vast knowledge, albeit often in narrow subject areas. Families fear that the loss of the specific Asperger’s diagnosis, as is the case with DSM-5, may result in the loss of specially tailored, individualized and, importantly, reimbursable, appropriate services for their children [10-13]. Serious concerns have been raised regarding the DSM-IV to −5 changes [14-19].

Although there are no agreed upon neuro-imaging criteria to diagnose ASP, there have been a number of studies that raise the potential for this possibility. In 2008, McAlonan *et al*. differentiated subjects with ASP and HFA on the basis of magnetic resonance imaging (MRI) differences in grey matter volumes [20], and in 2009 on the basis of differences in white matter volumes [21]. In 2011, Yu *et al*. differentiated ASP and ‘autism’ on the basis of grey matter volume: ‘Whereas grey matter differences in people with Asperger’s Syndrome compared with controls are sparser than those reported in studies of people with autism, the distribution and direction of differences in each category are distinctive’ [22]. However, the regions delineated by Yu *et al*. do not coincide completely with the regions defined by McAlonan *et al*. [20].

Comparisons between older ASP and HFA subjects have demonstrated better language and potentially differing brain anatomy and/or function within the ASP population [23-27]. Although these findings suggest that initial group differences of early language development - required for HFA by definition [2] - persist to later ages, they do not demonstrate that ASP and HFA subjects can be reliably differentiated. The findings suggest that ASP and HFA could be physiologically different entities but they do not distinguish between this possibility and the alternative possibility that the group differences may simply reflect differing degrees of the same basic underlying brain pathophysiology.

A known disease may constitute the tail end of a population distribution function or it may constitute a second, separable distribution of its own. Defining ASP as a separate entity from ASD might be as simple as defining a reliable, critical point on the ASD population distribution’s high functioning tail beyond which ASP is present and before which it is not. On the other hand, ASP may demonstrate a non-overlapping, separate distribution of its own. Recognition of complicated multimodal combinations of separate distributions is a complex statistical process [28,29].

The approach chosen in the current study was to determine whether there might be objective, unbiased, electrophysiological markers that can significantly distinguish ASP from ASD. For this determination EEG spectral coherence was chosen. EEG coherence represents the consistency of phase difference between two EEG signals (on a frequency by frequency basis) when compared over time and thus yields a measure of synchrony between the two EEG channels and an index of brain connectivity between the brain regions accessed by the chosen electrodes. High coherence represents a measure of strong connectivity and low coherence a measure of weak connectivity [30].

A great advantage of coherence is that it provides a quantifiable measure of between-region (electrode) connectivity that is essentially invisible to unaided visual inspection of raw EEG. There are at least three possible explanations for this phenomenon. First, coherence is calculated on a frequency by frequency (sine wave by sine wave) basis and EEG typically presents a complex and simultaneous mixture of many sine waves, each of a different frequency. Second, high coherence reflects a stable phase relationship (stable phase difference) between sine waves of the same frequency over time. The human eye is relatively poor in the visual assessment of phase shift stability over time, especially when many sine waves at multiple frequencies are simultaneously present as is the case in typical EEG. Furthermore, phase shift stability typically varies among differing spectral frequencies. Third, reliable and replicable coherence measures typically require relatively long EEG segments - minutes in length. These long epochs further confound an electroencephalographer’s ability to reliably estimate by unaided visual inspection the coherence between two channels of EEG. One of the best examples to graphically illustrate the difference between simple correlation and coherence in EEG was provided by Guevara and Corsi-Carbrera in 1996; however, the authors primarily utilized only simple sine wave segments for their explanatory illustrations [31].

Coherences among all possible electrodes and all frequencies produce thousands of variables. Principal components analysis (PCA) allows objective reduction of coherence data dimensionality to a much smaller number of statistically independent coherence factors, typically no more than 40, with minimal loss of information content [32-36]. Furthermore, PCA reduction of coherence data sets obviates the need to reduce data on the basis of *a priori* specified brain connectivity selections, and thus avoids the potential of investigator bias.

In 2012, the authors demonstrated that a stable pattern of EEG spectral coherence factors separated ASD subjects from neurotypical control subjects [36]. For this demonstration the two extremes of the ASD spectrum had been excluded from the ASD sample studied, namely HFA and ASP on one hand, and global developmental delay on the other. Subjects with Pervasive Developmental Disorder not otherwise specified (PDD-nos) were retained in the ASD sample. The resulting analyses conclusively demonstrated highly significant, reliable, stable classification success of neurotypical controls versus subjects with ASD on the basis of 40 coherence factors [36].

The first aim in this study was to test how a new independent ASP sample would be classified using discriminant rules that were developed on the 40 PCA-based EEG coherence factors that had previously, successfully distinguished subjects with ASD from neurotypical controls [36]. The second aim was to explore whether new EEG coherence-based classification rules could be derived to separate the ASP from the ASD population.

## Methods

All analyses were performed at the Boston Children’s Hospital (BCH) Developmental Neurophysiology Laboratory (DNL) under the direction of the first author. This laboratory maintains a comprehensive database of several thousand patients and research volunteers including unprocessed (raw) EEG data in addition to referral information. Patients typically are referred to rule out epilepsy and/or sensory processing abnormalities by EEG and evoked potential study. Only EEG data are utilized and reported in this study.

### Patients with autism spectrum disorders and with Asperger’s syndrome

The goal of the current study was to select only those patients, ranging in age from 2 to 12 years, diagnosed by experienced clinicians as having ASD or ASP. Excluded were all subjects with co-morbid neurological diagnoses that might exert an independent and confounding impact upon EEG data.

The inclusion criteria for ASD and the ASP groups consisted of an age of 2 to 12 years and a disorder diagnosis, as determined by an independent child neurologist, psychiatrist or psychologist specializing in childhood developmental disabilities at BCH or at one of several other affiliated Harvard teaching hospitals. Diagnoses relied upon DSM-IV [2], Autism Diagnostic Interview, revised (ADI-R) [37] and/or Autism Diagnostic Observation Schedule (ADOS) [38,39] criteria, aided by clinical history and expert team evaluation. All clinical diagnoses were made or reconfirmed within approximately one month of EEG study, thereby obviating diagnostic variation related to time from diagnosis to EEG assessment, a recently recognized important issue [40,41].

Exclusion criteria for both ASD and ASP were: (1) co-morbid neurologic syndromes that may present with autistic features (for example, Rett’s, Angelman’s and fragile X syndromes and also tuberous sclerosis and mitochondrial disorders); (2) clinical seizure disorders or EEG reports suggestive of an active seizure disorder or epileptic encephalopathy such as the Landau-Kleffner syndrome (patients with occasional EEG spikes were not excluded); (3) a primary diagnosis of global developmental delay or developmental dysphasia; (4) expressed doubt by the referring clinician as to the clinical diagnosis; (5) taking medication(s) at the time of the study; (6) other concurrent neurological disease processes that might induce EEG alteration (for example, hydrocephalus, hemiparesis or known syndromes affecting brain development); and (7) significant primary sensory disorders, for example, blindness and/or deafness.

A total of 430 subjects with ASD met the above study criteria and were designated as the study's ASD sample. For further detailed sample description see Duffy and Als [36]. A total of 26 patients met the above study criteria for ASP and were designated as the study's ASP sample.

### Healthy controls

From among normal (neurotypical) children recruited and studied for developmental research projects, a comparison group of children was selected as normally functioning, while avoiding creation of an exclusively 'super-normal' group. For example, subjects with the sole history of prematurity or low-weight birth and not requiring medical treatment after birth hospital (Harvard affiliated hospitals) discharge were included.

Necessary inclusion criteria were age between 2 and 12 years corrected for prematurity (as indicated), living at home and identified as functioning within the normal range on standardized developmental and/or neuropsychological assessments performed in the course of the respective research study.

Exclusion criteria were as follows: (1) Diagnosed neurologic or psychiatric illness or disorder or expressed suspicion of such, for example, global developmental delay, developmental dysphasia, attention deficit disorder and attention deficit with hyperactivity disorder; (2) abnormal neurological examination as identified during the research study; (3) clinical seizure disorder or EEG report suggestive of an active seizure disorder or epileptic encephalopathy (individuals with rare EEG spikes again were not excluded); (4) noted by the research psychologist or neurologist to present with ASD or ASP features; (5) newborn period diagnosis of intraventricular hemorrhage, retinopathy of prematurity, hydrocephalus or cerebral palsy, or other significant conditions likely influencing EEG data; and/or (6) taking medication(s) at time of EEG study.

A total of 554 patients met the criteria for neurotypical controls and were designated as the study's control sample. For further description of the control sample see Duffy and Als [36].

### Institutional review board approvals

All neurotypical control subjects and their families gave informed consent, and assent as age appropriate, in accordance with protocols approved by the Institutional Review Board, Office of Clinical Investigation of BCH, in full compliance with the Helsinki Declaration. Subjects with ASD or ASP, who had been referred clinically, were studied under a separate BCH Institutional Review Board protocol, also in full compliance with the Helsinki Declaration, which solely required de-identification of all personal information related to the collected data without requirement of informed consent.

### Measurements and data analysis

#### EEG data acquisition

**Figure 1:**
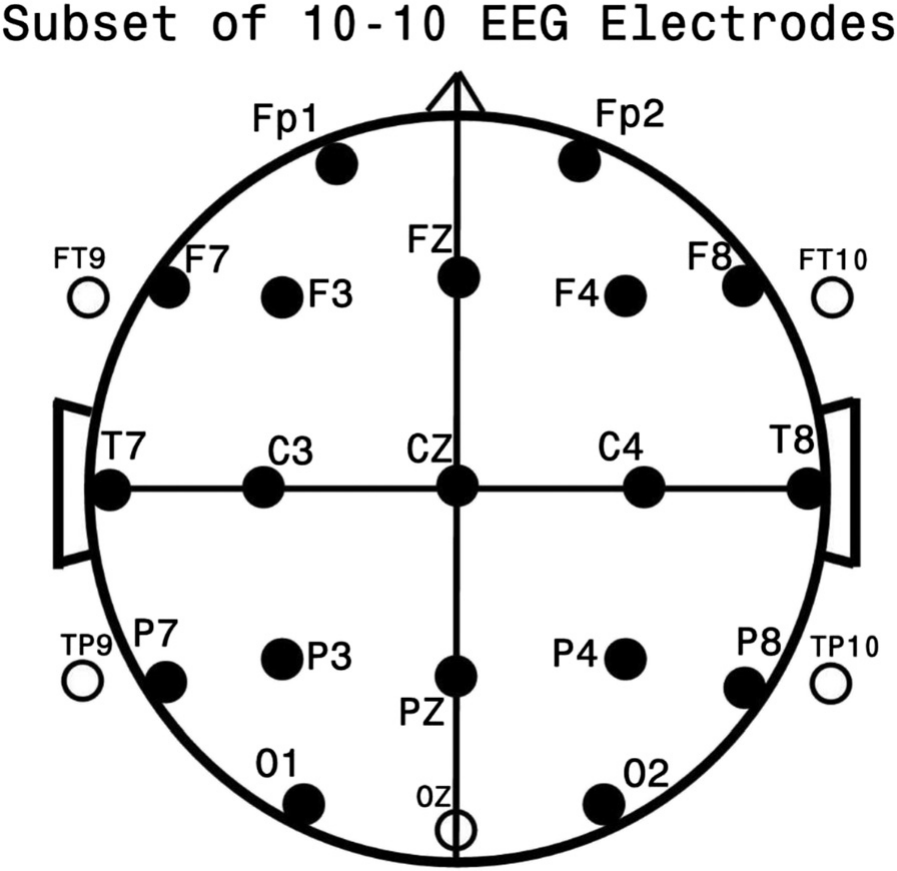
**Standard EEG electrode names and positions. **Head in vertex view, nose above, left ear to left. EEG electrodes: Z: Midline; FZ: Midline Frontal; CZ: Midline Central; PZ: Midline Parietal; OZ: Midline Occipital. Even numbers, right hemisphere locations; odd numbers, left hemisphere locations: Fp: Frontopolar; F: Frontal; C: Central; T: Temporal; P: Parietal; O: Occipital. The standard 19, 10–20 electrodes are shown as black circles. An additional subset of five, 10–10 electrodes are shown as open circles. Reprinted from Duffy FH and Als H with permission [36].

Registered EEG technologists, naïve to the study's goals, and specifically trained and skilled in working with children within the study's age group and diagnostic range, obtained all EEG data for the study from 24 gold-cup scalp electrodes applied with collodion after measurement: FP1, FP2, F7, F3, FZ, F4, F8, T7, C3, CZ, C4, T8, P7, P3, PZ, P4, P8, O1, OZ, O2, FT9, FT10, TP9, TP10 (see Figure 1). EEG data were gathered in the awake and alert state assuring that a minimum of eight minutes of waking EEG was collected. Data were primarily gathered with Grass™ EEG amplifiers with 1 to 100 Hz band-pass filtering and a 256 Hz sampling rate (Grass Technologies Astro-Med, West Warwick, RI, USA). One other amplifier type was utilized for five patients with ASD (Bio-logic™; Bio-logic Technologies, San Carlos, CA, USA; 250 Hz sampling rate, 1 to 100 Hz band-pass), and one other amplifier type was utilized for 11 control subjects (Neuroscan™; Compumedics Neuroscan, Charlotte, NC, USA; 500 Hz sampling rate, 0.1 to 100 Hz band-pass). Data from these two amplifiers, sampled at other than 256 Hz, were interpolated to the rate of 256 Hz by the BESA 3.5™ software package (BESA GmbH, Gräfelfing, Germany). As the band-pass filter characteristics differed among the three EEG machines, frequency response sweeps were performed on all amplifier types to permit modification of data recorded to be equivalent across amplifiers. This was accomplished by utilizing special software developed in-house by the first author using forward and reverse Fourier transforms [42].

#### *Measurement issues*

EEG studies are confronted with two major methodological problems. First is the management of the abundant artifacts, such as eye movement, eye blink and muscle activity, observed in young and behaviorally difficult to manage children. It has been well established that even EEGs that appear clean by visual inspection may contain significant artifacts [43,44]. Moreover, as shown in schizophrenia EEG research, certain artifacts may be group specific [45]. Second is capitalization upon chance, that is, application of statistical tests to too many variables and subsequent reports of chance findings in support of an experimental hypothesis [43,46]. Methods discussed below were designed to specifically address these two common problems.

#### *1. Artifact management*

As previously outlined in greater detail [36], the following steps were instituted for artifact management:

(1) EEG segments containing obvious movement artifact, electrode artifact, eye blink storms, drowsiness, epileptiform discharges and/or bursts of muscle activity were marked for removal from subsequent analyses by visual inspection.

(2) Data were subsequently filtered below 50 Hz with an additional 60 Hz mains filter.

(3) Remaining lower amplitude eye blink was removed by utilizing the source component technique [47,48], as implemented in the BESA software package. These combined techniques resulted in EEG data that appeared largely artifact free, with rare exceptions of low level temporal muscle fast activity artifact and persisting frontal and anterior temporal slow eye movements, which remain, none-the-less, capable of contaminating subsequent analyses.

(4) A regression analysis approach [49] was employed to remove these potential remaining contaminants from subsequently created EEG coherence data. Representative frontal slow EEG spectral activity representing residual eye blink and representative frontal-temporal EEG spectral fast activity representing residual muscle artifact were used as independent variables within multiple regression analysis, where coherence variables were treated as dependent variables. Residuals of the dependent variables, now uncorrelated with the chosen independent artifact variables, were used for the subsequent analyses.

#### *2. Data reduction - calculation of spectral coherence variables*

Approximately 8 to 20 minutes of awake state, artifact free, EEG data per subject were transformed by use of BESA software, to the scalp Laplacian or current source density (CSD) estimates for surface EEG studies. The CSD technique was employed as it provides reference independent data that are primarily sensitive to underlying cortex and relatively insensitive to deep/remote EEG sources, and minimizes the effect of volume conduction on coherence estimates by emphasizing sources at smaller spatial scales than unprocessed potentials. This approach obviates coherence contamination from reference electrodes and minimizes contaminating effects from volume conduction [30,50].

Spectral coherence was calculated, using a Nicolet™ software package (Nicolet Biomedical Inc., Madison, WI, USA) according to the conventions recommended by van Drongelen [51] (pages 143–144, equations 8.40, 8.44). Coherency [52] is the ratio of the cross-spectrum to the square root of the product of the two auto-spectra and is a complex-valued quantity. Coherence is the square modulus of coherency, taking on a value between 0 and 1. In practice, coherence is typically estimated by averaging over several epochs or frequency bands [51]. A series of two-second epochs was utilized over the total available EEG segments. Spectral coherence utilizing 24 channels and 16, 2 Hz wide spectral bands from 1 to 32 Hz, results in 4,416 unique coherence variables per subject, purged of residual eye movement and/or muscle artifact by regression as explained above. The data processing described above was used in the current as well as our prior study of ASD [36].

#### *3. Creation of 40 coherence factors*

Forty coherence factors had been created utilizing PCA with Varimax rotation prior to this study from the 4,416 coherence variables per subject individual of the independent study population consisting of the combined neurotypical controls and subjects with ASD [36]. The 40 factors described over 50% of the total variance within that combined population. These 40 coherence factors were created in the current study for each individual of the new sample of 26 subjects with ASP. The inherently unbiased data reduction by PCA eliminated capitalization on chance and investigator selection bias.

### Data analysis

The BMDP2007™ statistical package (Statistical Solutions, Stonehill Corporate Center, Saugus, MA, USA) [53] was utilized for all standard statistical analyses with the exception of PCA (see above and [36]).

### Discrimination of groups by EEG spectral coherence data

Program 7M was used for two-group discriminant function analysis (DFA) [54-56]. Program 7M produces a new canonical variable, the discriminant function, which maximally separates two groups based on a weighted combination of entered variables. DFA defines the significance of a group separation, summarizes the classification of each participant, and provides an approach for the prospective classification of individuals not involved in discriminant rule generation or for classification of a new population. The analysis reports the significance of group separation statistically by Wilks’ lambda with Rao’s approximation. To estimate prospective classification success, the jackknifing technique, also referred to as the leaving-one-out process, was used [57,58]. By this method, discriminant function is formed on all individuals but one. The left-out individual is subsequently classified. This initial left out individual is then folded back into the group (hence ‘jackknifing’), and another individual is left out. The process is repeated until each individual has been left out and classified. The measure of classification success is then based upon a tally of the correct classifications of the left-out individuals.

### Assessment of population distribution

The samples’ distribution characteristics were described by Program 2D. It incorporates the standard Shapiro-Wilk or W-test of normality for large samples, considered to be an objective and powerful test of normality [59,60]. It also calculates skewedness, a measure of asymmetry with a value of zero for true symmetry, and a standard error (value/SE). Positive numbers above +2.0 indicate skew to the right and below −2.0 skew to the left. In addition, the W-test calculates kurtosis, a measure of long-tailedness. The tail-length value of a true normal tail is 0.0. If the tail length, value/SE, is above +2.0, the tails are longer than for a normal distribution, and if it is below −2.0, the tails are shorter than for a true normal distribution.

Muratov and Gnedin recently described two relatively new techniques that search for bimodality within a given population distribution [29]. Gaussian mixture modeling determines whether the population deviates statistically from unimodality. It also searches for all potential underlying bimodal populations and determines the significance of the best possible bimodal solution. These authors also described the Dip test [61], which statistically compares the actual population distribution with the best possible unimodal distribution to look for flat regions or dips between peaks as would be found in bimodally distributed populations.

### Multiple regression program

Program 6R facilitates the multivariate prediction of a single dependent variable on the basis of a set of selected independent predictor variables. The program calculates a canonical variable formed from a rule-based linear combination of independent variables, which predict the independent variable. Program 6R was used for prediction of coherence measures from multiple EEG spectral measures sensitive to known EEG artifacts (for example, temporal muscle fast beta and frontal slow delta eye movement). The fraction of a coherence measure that was predicted by artifact was removed and the ‘residual’ coherence measures were subsequently utilized as variables, now uncorrelated with any known artifact signal.

## Results

### Asperger’s syndrome classification as control or autism spectrum disorders

The 26 new subjects with ASP had a mean age of 7.07 years with a range from 2.79 to 11.39 years and consisted of 18 males and 8 females (male to female ratio of 2.25:1), comparable in age and gender distribution to the previously studied neurotypical control and ASD groups [36]. The 26 subjects with ASP and the populations of 554 controls and 430 subjects with ASD were submitted to a two-group DFA with the 40 coherence factors as input variables. The ASP subjects were designated to be passively classified on the basis of rules generated to differentially classify the control and ASD groups. As shown in Table 1, 96.2% of the ASP group (25 out of 26) were classified as belonging within the ASD group, and just 3.8% (1 out of 26) were classified as belonging within the control group. Factor 15 was the highest loading variable, that is, the first coherence factor chosen, on the discriminant function. Thus, within a neurotypical control versus ASD dichotomy, ASP subjects were securely classified as belonging to the ASD population.

**Table 1:** Discriminant analysis of control versus autism spectrum disorders; Asperger’s syndrome classified passively

**Variables utilized = 23**	**Percent correct**	**Control**	**ASD**
**First 4: Fac15, Fac17, Fac2, Fac16**			
Control	92.6	513	41
ASD	84.8	65	365
ASP	96.2^a^	1	25

Significance of group separation reported statistically:

Wilks’ lambda = 0.4991; F = 41.885; degrees of freedom = 23, 960; *P* ≤0.0001.

^a^Passively classified.

*ASD* Autism Spectrum Disorders, *ASP* Asperger’s Syndrome.

### Asperger’s syndrome classification as within or separate from autism spectrum disorders

An additional two-group DFA was performed comparing the new ASP (n = 26) population with the ASD population (n = 430), again with 40 coherence factors as input variables. The overall classification, as Table 2 shows, was highly significant (F = 6.05; degrees of freedom =16,439; *P* ≤0.0001). Jackknifing techniques correctly classified 92.3% of the patients with ASP (24 out of 26) and 84.4% of the patients with ASD (363 out of 430). Thus the coherence factors separated the ASP population from the ASD population with excellent classification success.

**Table 2:** New discriminant analysis asperger’s syndrome versus autism spectrum disorders, controls excluded

**Variables utilized = 16**	**Percent correct**	**ASD**	**ASP**
**First 4: Fac15, Fac3, Fac33, Fac40**			
**Remaining 12: Facs 9, 32, 1, 4. 6, 5, 21, 39, 10, 16, 25, 38**			
ASD	84.4	363	67
ASP	92.3	2	24

Significance of group separation reported statistically**:**

Wilks’ lambda = 0.81894; F = 6.045; degrees of freedom = 16, 439; *P* ≤0.0001.

*ASD* Autism Spectrum Disorders, *ASP* Asperger’s Syndrome.

**Figure 2:**
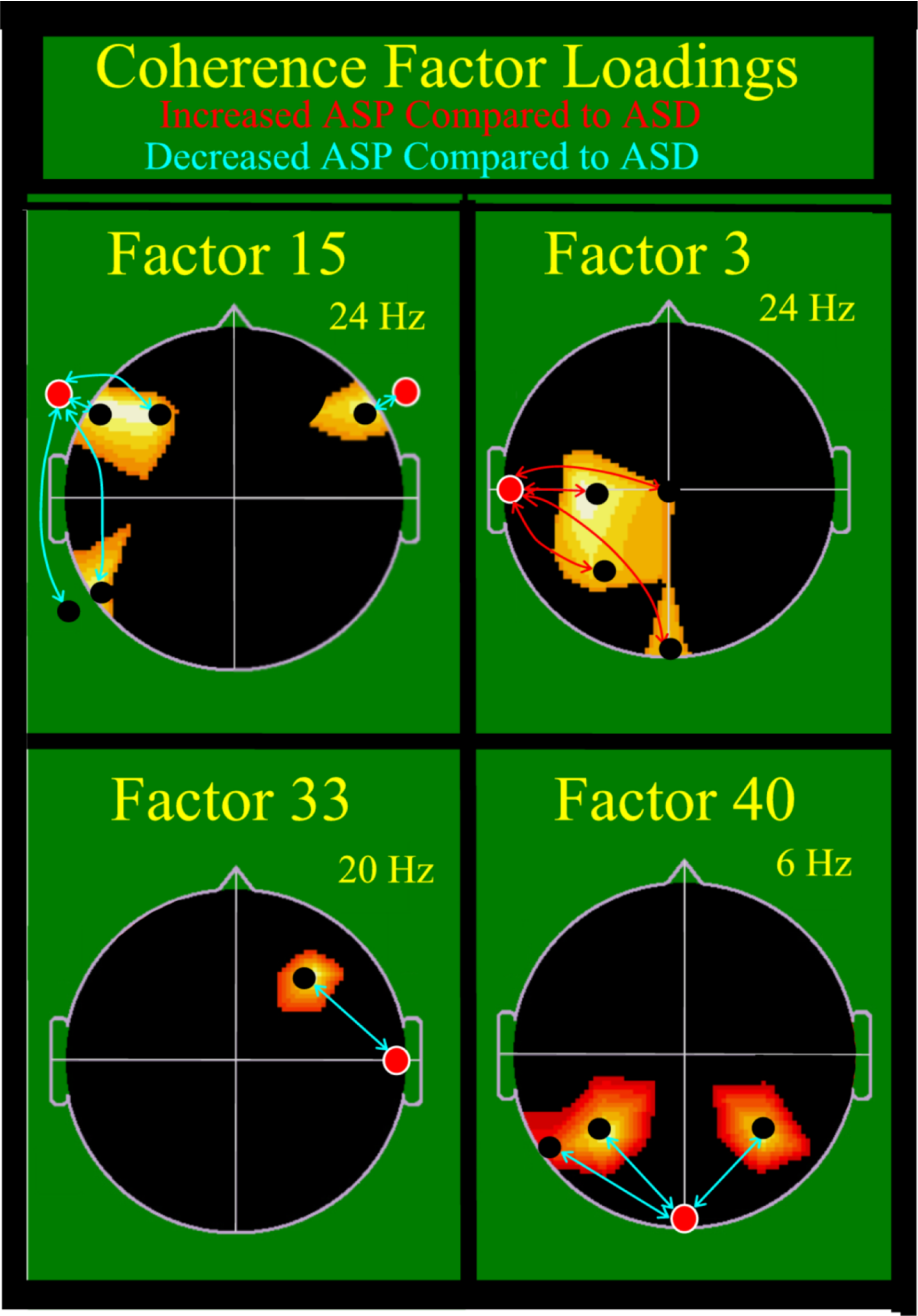
**Coherence loadings: four factors best differentiate Asperger’s syndrome from autism spectrum disorders.** EEG coherence factor loadings shown. View from above head, nose at top of each head image, left ear to left of image. Factor number is above each head and peak frequency for factor in Hz is above to right. Lines indicate top 85% coherence loadings per factor. Bidirectional color arrows delineate electrode pairs involved in the displayed factor. Red line = increased coherence in ASP group; blue-green line = decreased coherence in ASP group compared to ASD group. Relevant electrodes (see Figure 1) per factor are shown as black dots. The comparison electrode is shown as a red circle. Background colored areas are regions delineated by original PCA. Involved electrodes: Symbol ‘**↔**’ connects coherent electrodes for each factor Factor 15: FT9 **↔** TP9, F7, F3, P7 and FT10 ↔ F8; Factor 3: T7 **↔** C3, P3, CZ, OZ Factor 33: T8 **↔** F4 Factor 40: OZ **↔** P3, P7, P4. ASD, Autism Spectrum Disorders; ASP, Asperger’s Syndrome.

As Table 2 and Figure 2 illustrate, Factor 15 again was the first coherence factor chosen for the ASD-ASP discrimination. Factor 15 similarly had been the first factor chosen for most of the control versus ASD population discriminations in the prior study [36]. This factor indicates a reduced coherence between the left anterior and posterior frontal-temporal regions, and to a lesser degree between the right anterior temporal-frontal regions, for the ASP group compared with the ASD group. In contrast, the loading of the next factor chosen, Factor 3, demonstrated enhanced coherence between the left mid temporal region and the left central, parietal and occipital regions for the ASP group compared with the ASD group. The loadings of the next two factors selected, Factor 33 and Factor 40, demonstrated reduced right temporal-frontal coherence and reduced occipital to bilateral parietal coherence for the ASP compared with the ASD group. These first four were the most important factors; their coherence loading patterns are depicted in Figure 2. Twelve additional factor designations are also provided; their loading patterns are depicted and discussed in a previous publication [36].

Five subsamples, each consisting of 26 subjects with ASD, were randomly selected from the larger ASD population. The DFA process was repeated to determine whether these randomly selected subsets of subjects with ASD could be classified as separate from the remaining ASD population. As Table 3 shows, jack-knifed classification success for the five random sets averaged just 48.5%, that is, below the chance level of 50%. None of the five DFA demonstrated significant Wilks’ lambda. Note that the list of chosen factors did not include Factor 15 as had been selected first in the current and prior analyses. Note, also, that there is a lack of consistency in factor selection among the five-group analyses. Thus, random samples of 26 subjects with ASD were not significantly and reliably separable by discriminant analysis from the remaining ASD population.

**Table 3:** Discriminant analysis of five groups of 26 patients with autism spectrum disorder versus the remaining 404 subjects in that population

**Groups of 26**	**Percent correct**	**As ASD 26**	**As group of 404**	**Total: factors used**
1	50.0	13	13	6: 23, 8, 38, 35
2	53.8	14	12	16: 28,10,32,14
3	46.2	12	14	2: 33, 19
4	50.0	13	13	3: 38, 40, 11
5	42.3	11	15	4: 2, 22, 20, 13
Average	48.5			

### Asperger’s syndrome population, tail of the autism spectrum disorders distribution curve or separate population?

The distribution characteristics of the canonical variable defined by the DFA separating the ASP from the ADS groups were described for each sample separately. The ASD population distribution parameters were as follows: normality statistic, W = 0.9881, *P* = 0.8375; skewedness statistic, W = 0.03, value/SE = −0.0265; kurtosis statistic, W = 1.35, value/SE = 5.728. This indicated that the ASD sample was found to be within the limits of a normal distribution, was symmetrical, and had somewhat longer tails than the typical normal distribution, not unusual for a clinical population. All five randomly selected subsets of the ASD population also demonstrated normal distributions as anticipated by statistical theory [62].

**Figure 3:**
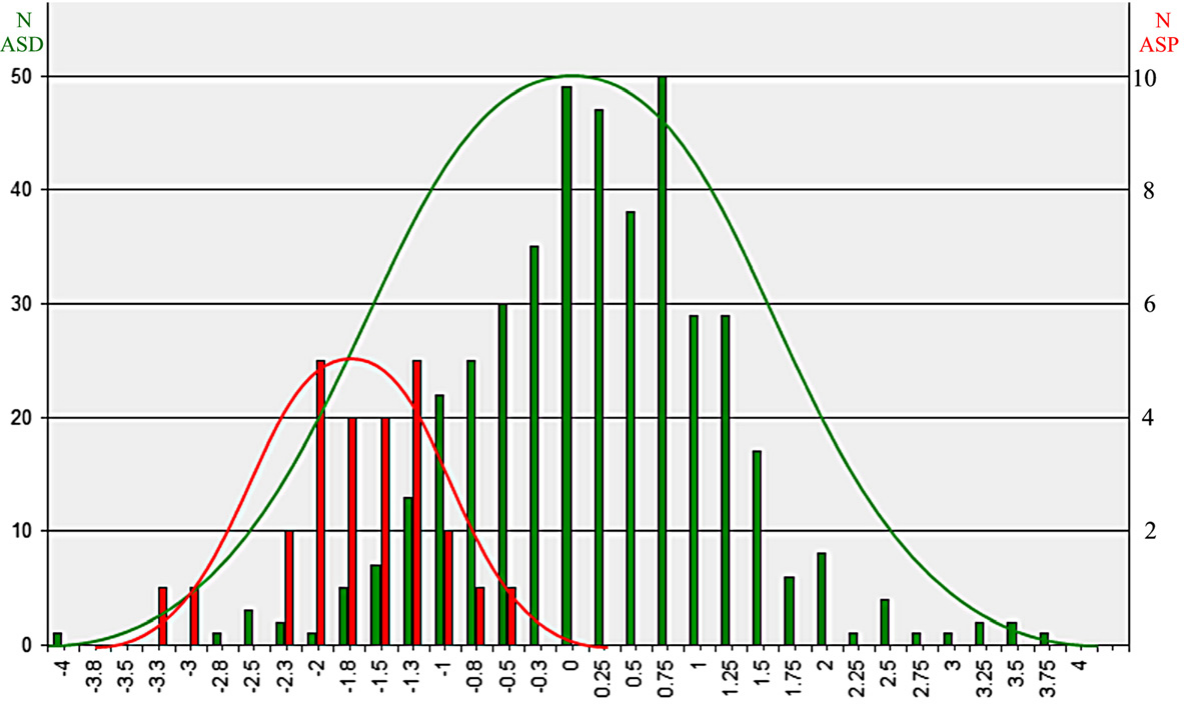
**Asperger’s syndrome and autism spectrum disorders population distributions. **Population distribution histograms are shown for the ASD (green, n = 430) and ASP (red, n = 26) groups. The horizontal axis is the discriminant function value developed to differentiate the ASD and ASP groups on the basis of coherence variables. It varies from −4.0 to +4.0 units. The histograms are formed from bins 0.25 units wide. The populations are both Gaussian in distribution. A smoothed Gaussian distribution is shown above the true histogram data distribution as estimated by Excel software. Discriminant analysis significantly separates the two groups. The ASP population is displayed on an expanded vertical scale. ASD, Autism Spectrum Disorders; ASP, Asperger’s Syndrome.

The new sample of 26 subjects with ASP showed distribution parameters as follows: normality statistic, W = 0.9606, *P* = 0.4222; skewedness statistic, W = −0.61, value/SE = −1.281; kurtosis statistic, W = 0.33, value/SE = 0.347. This indicated that the ASP sample distribution was also within the limits of a normal population, was symmetrical, and had tails that conformed to expected lengths (see Figure 3) and was therefore characterized as Gaussian normal.

When the ASD and ASP populations were combined and displayed (Figure 3), the ASP population appeared as a small Gaussian distribution in the left end of the ASD population. However, the Gaussian mixture modeling process indicated that the best bimodal means, nevertheless, were close and did not differ statistically. The Dip test similarly indicated that the probability for a deviation from unimodality was not significant.

## Discussion

The goal of this study was to explore the relationship between a sample of subjects clinically defined as having ASP, and a population of previously well-studied neurotypical controls and subjects with ASD. The dependent variables of interest, detailed in a prior study [36], were 40 EEG coherence factors derived from systematically de-artifacted EEG data.

### Specific goals and findings

The study’s first goal was to determine how a previously defined and statistically validated discriminant function, developed to classify individuals as belonging to a control or an ASD population, would classify subjects with ASP, whose data had not influenced the derivation of the discriminant function. Results (Table 1) showed that the control versus ASD discriminant function classified 25 of 26 patients with ASP (96.2%) as belonging to the ASD sample. This indicates that subjects with ASP are neurophysiologically closer to the ASD population than to the neurotypical control population.

The study’s second goal was to determine if the 26 subjects with ASP were, nonetheless, systematically separable from the larger population of 430 subjects with ASD. Using DFA, the subjects with ASP were indeed significantly separated (*P* ≤0.0001) from the ASD population; 92.3% (24 out of 26) of those with ASP were classified as ASP rather than as ASD. These results show that subjects with ASP, although associated with the broader autism spectrum population, manifested significant physiological differences in EEG connectivity (as measured coherence factors) to distinguish them from the subjects with ASD. To test whether this subsample separation was a random result, that is, whether a randomly chosen subsample of individuals could also be classified as a distinct subgroup, five randomly selected sets of 26 subjects with ASD were also compared by DFA to the remaining ASD population. The average classification success was 48.5%, that is, less than chance; the highest classification success reached was 53.8%. These results suggest that the ASP subgroup discrimination from the larger ASD group was not the result of sampling artifact but in fact due to true group differences, because the findings held for the ASP separation but not for the ASD subsample discrimination attempts.

The pattern of coherence difference, as shown by the loading patterns depicted in Figure 2 (Factor 15), demonstrated that the ASP population showed even more reduction of left lateral anterior-posterior coherence than the ASD group. This was an unexpected finding as Factor 15 was postulated to be a language-related factor based upon its similarity to the spatial location of the Arcuate Fasciculus [36], and subjects with ASP typically have better language function than do those with ASD. The solution to this unanticipated finding became clearer through inspection of the Factor 3 coherence loadings, which showed that the ASP group demonstrated markedly increased left mid temporal to central parietal-occipital coherence. It is speculated that Factor 3’s broadly increased left temporal connectivity may partially compensate for the language deficiency suggested by Factor 15, potentially facilitating acquisition of language skill in ASP without significant developmental delay. It is also proposed that the postulated compensation may not completely facilitate all aspects of normal language development, and may result in the several, readily identifiable, higher level differences of language use observed in subjects with ASP. Examples include excessive pedantic formality, verbosity, literal interpretation devoid of nuance and prosodic deficiency, to name a few [63]. The final two factors chosen, Factors 33 and 40, show a pattern of reduced coherence loadings in the ASP group that may correspond to differences in visual-spatial functioning and right hemispheric characteristics that have been described as part of the lack of social nuance and special kind of ‘oblivious to context’ personality characteristics observed in individuals with ASP [64,65].

The study’s third goal was to determine whether the subjects with ASP represent a tail of the ASD population distribution or a distinct population. Inclusion of the ASP to the ASD population (Figure 3) did not result in a statistically significant bimodal distribution as would be seen if the ASD and ASP populations represented completely differing clinical entities. However, the asymmetrically high ASD/ASP population ratio of 16.5:1 was above the maximally tested ratio of 10:1 for the Gaussian mixture modeling and Dip tests employed [29]; typical ratios are 3 or 4 to 1. The small size of the tested ASP population limits definitive determination of whether ASP is a separate entity to ASD. Study of a larger ASP population is necessary to asses this important question in a more conclusive manner. Nevertheless, it is striking that the relatively small sample of 26 randomly referred subjects with ASP manifested a normal Gaussian distribution as opposed to one demonstrating an asymmetrical distribution as might be expected if the sample simply constituted subjects non-randomly selected from the high functioning end of the ASD population curve. At this point, current study results are consistent with ASP forming one end of the ASD population. This is similar to the demonstration by Shaywitz *et al*. that reading disability represents the ‘low end tail’ of the reading ability curve and not a distinctly separate population [66].

Additional questions concern the portion of the ASD population distribution that overlapped with the ASP population distribution (Figure 3), including the 69 individual misclassifications within the ASD versus ASP discriminant analysis (Table 2). The population overlap may represent clinical misdiagnoses or constitute noise within the statistical classification process. Alternatively, the population overlap may indicate that HFA and ASP are the same physiological entity. Indeed, it has been clinically observed that the diagnosis of ASP by DSM-IV criteria [2] may be obscured by poor reliability in a family’s recollection of early language delay or by the belief of some clinicians that the diagnosis of ASP should be made on the basis of the patient’s current behavioral profile without weighting the presence or absence of early language delay. ASP and HFA are often spoken of, especially by neurologists, as a single entity or at least closely related entities.

The limitation of the small ASP sample size is the main drawback of the current study. A larger prospective study must be conducted to address whether - separately or together - ASP significantly differs neurophysiologically from ASD, and whether ASP and HFA constitute single or separable populations.

Although the findings above in many ways agree with the DSM-5 [9] placement of ASP within the broad autistic spectrum, they also demonstrate that patients with ASP can be physiologically distinguished from those with ASD. Recognition of ASP as a separate entity is important from the patients’ perspectives of obtaining appropriate medical and educational services as well as of establishing a personal identity. As an example of the latter, the well-read author with Asperger’s Syndrome, J E Robinson [67], reported in a televised interview that it ‘was life changing … ’ to discover as an adult that he had a known, named syndrome and that ‘ … there were so many people like me.’

## Conclusion

A diagnostic classifier based upon EEG spectral coherence data, previously reported to accurately classify controls and ASD subjects [36], has identified ASP subjects as within the ASD population. Thus, there is justification to consider Asperger’s Syndrome as broadly belonging within the Autism Spectrum Disorders. However, there is also evidence demonstrating that ASP subjects can be physiologically distinguished from ASD subjects. Just as dyslexia is now recognized as the low end tail of the reading ability distribution curve [63], so Asperger’s Syndrome may be similarly and usefully defined as a distinct entity within the higher functioning tail of the autism distribution curve. Larger samples are required to determine whether ASP subjects should be considered as an entity physiologically distinct from the ASD population or whether they form an identifiable population within the higher-functioning tail of ASD.

EEG spectral coherence data, as presented, provide easily obtained, unbiased, quantitative, and replicable measures of brain connectivity differences relevant to these issues.

## Abbreviations

ASD: Autism spectrum disorder; ASP: Asperger’s syndrome; BCH: Boston Children’s Hospital; DFA: Discriminant function analysis; DSM: Diagnostic and statistical manual of mental disorders; EEG: Electroencephalogram, electroencephalography, electroencephalographic; HFA: High functioning autism; PCA: Principal components analysis; SE: Standard error.

## Competing interests

The authors declare that they have no competing interests.

## Authors’ contributions

Study concept and design, and interpretation of the results was performed by all authors. FHD and AS selected clinical patients to be illustrated. FHD and HA selected specific neurotypical controls. FHD was responsible for the acquisition and preparation of neurophysiologic data. FHD and GBM performed the statistical analyses. FHD had full access to all the data in the study and takes responsibility for all aspects of the study including integrity of data accuracy and data analysis. All authors collaborated in writing and editing the paper and approved the final manuscript.

## Authors’ information

FHD: Physician, child neurologist, clinical electroencephalographer and neurophysiologist with undergraduate degrees in electrical engineering and mathematics. Current research interests are in neurodevelopmental disorders and epilepsy, including the development and utilization of specialized analytic techniques to support related investigations. AS: Cognitive neuroscientist with specialized interests in the neurophysiological identification of neurodevelopmental disorders, particularly developmental language disorders. GBM: Neuropsychologist and statistician with interests in pediatric neurodevelopment. HA: Developmental and clinical psychologist with research interests in newborn, infant and child neurodevelopment including generation of early predictors of later outcome from behavioral, magnetic resonance imaging and neurophysiologic data.
